# Bibliometric Analysis and Review of Global Academic Research on Drug Take-Back Programs

**DOI:** 10.3390/healthcare13212711

**Published:** 2025-10-27

**Authors:** Shuzhe Wu, Xi Zhou, Xianmin Hu, Jun Wang

**Affiliations:** Department of pharmacy, College of Medicine, Wuhan University of Science and Technology, Wuhan 430065, China

**Keywords:** drug take-back program, bibliometric analysis, unwanted medication disposal, Web of Science, CiteSpace, VOSviewer

## Abstract

**Background/Objectives**: As safe, eco-friendly, and legally compliant solutions for the disposal of unwanted medications, drug take-back systems have attracted extensive research attention. However, there is a lack of systematic mapping of global trends, collaborative networks, research themes, and hotspots in this field. This study aimed to conduct a comprehensive bibliometric analysis and review of global academic research on drug take-back programs. **Methods**: Peer-reviewed research articles on drug take-back programs, published between 2005 and 2025, were retrieved from the Web of Science Core Database. Microsoft Office Excel 2019, VOSviewer (v.1.6.17), and CiteSpace (v.6.1.R3 Advanced) were used to assess publication/citation trends, countries, institutions, authors, journals, disciplines, references, and keywords. Narrative analysis was employed to synthesize data from the included articles and identify core research themes. **Results**: A total of 149 eligible articles with 4520 citations were included, involving 619 authors, 52 countries/regions, 310 institutions, and 95 journals. Publication/citation counts increased significantly between 2005 and 2025. The United States led in both publication output and collaborative research; Mercer University was the most influential institution, but international and cross-institutional collaboration remained limited. Environmental Sciences ranked first among disciplinary categories in drug take-back research, followed by Pharmacology/Pharmacy. Core research themes underpinning this field included stakeholders’ knowledge–attitude–practice assessment (76 articles), returned medication treatment (37 articles), intervention evaluation (25 articles), policy analysis (7 articles), and the role of drug take-back programs in mitigating environmental and public health hazards caused by medicine wastes (4 articles). **Conclusions**: Scholarly attention to drug take-back programs has grown steadily. Future research should prioritize cross-sectoral and international cooperation, develop and adopt evidence-based interventions to optimize the safety, sustainability, and accessibility of drug take-back systems on a global scale.

## 1. Introduction

With the increasing growth of the global pharmaceutical market, storing medications at the household level has become a common practice [1,2,3]. Many families, particularly those with children and/or caring for individuals with chronic illnesses, tend to keep excess pharmaceutical products at home for ‘just in case’ future use [4]. A previous systematic review concluded that approximately 77% of households worldwide store medications [5]. A community-based cross-sectional study in Vietnam surveyed 800 households and found a high proportion (71.6%) kept unused medicines [6]. Another study in Burdur, Türkiye, showed that among pharmaceuticals prescribed to the general public participants, only 50.6% were used, with the remaining 49.4% being unused and stored at home [7]. In China, household storage of common medications, such as anti-cold, gastrointestinal, and analgesic agents, is also prevalent, especially among urban families [8,9,10], and it has been reported that approximately 78.6% of Chinese families tend to store medications [8].

However, usually not all medications stored at home are fully used, due to diverse factors such as expiration, patient noncompliance, forgetfulness, early improved medical conditions, excessive or altered prescriptions, and patient demise [2,6,11]. This leads to a buildup of unused medications in households, creating a major challenge for the proper disposal of unwanted medicines. If these unwanted medications are not disposed of correctly, expired or unused drugs at home pose significant health risks to family members, including improper self-medication, intoxication, and prescription drug abuse [12]. Surveys consistently show that the general public most commonly disposes of unwanted medications by throwing them in the garbage or flushing them down sinks or toilets [13,14,15]. However, drug waste in garbage can cause accidental drug exposure in children, pets, and wild animals [12]. Flushing disposal allows active pharmaceutical ingredients to enter the sewage system as emerging contaminants [11,16]. Current wastewater and solid waste treatment processes are often ineffective at fully removing pharmaceutical residues, enabling these contaminants to continuously enter receiving environmental systems [16,17,18]. To date, over 992 active pharmaceutical ingredients have been frequently detected as contaminants in various environmental samples worldwide, including surface water, groundwater, soils, sediments, and drinking water [19]. As biologically active substances custom-designed to be persistent and alter biochemical pathways even at low doses, pharmaceuticals may pose potential risks to non-targeted organisms, biodiversity, and the entire ecosystem, even at trace residual concentrations in environmental matrices [11,17,20].

Therefore, the appropriate and safe disposal of unwanted medications via drug take-back programs has been widely recognized as a global issue of concern [3,21,22,23]. The European Union (EU) adopted mandatory legal provisions in 2004, explicitly requiring all Member States to establish “an appropriate collection system” for unused and/or expired pharmaceuticals. However, it did not set unified regulations for specific collection infrastructure (e.g., collection point setup) or funding raising and allocation mechanisms, allowing Member States to implement the program independently [21]. In the United States (US), the Drug Enforcement Administration (DEA) outlined procedures for enforcing the federal drug disposal statute in 2014, covering authorized collectors, collection centers, and collection methods [3]. The Food and Drug Administration (FDA) also issued guidance recommending the public return unused pharmaceuticals to certified take-back locations like health centers and retail pharmacies [24,25]. US state-level laws differ in accepted medication types, collection site arrangements, and funding mechanisms. By 2022, one-third of states will provide financial support for drug take-back systems, and over 25 states have enacted Extended Producer Responsibility regulations mandating pharmaceutical manufacturers cover the costs of local take-back initiatives [3]. As early as 1998, Australia launched the national “Return Unwanted Medicine (RUM) Project” directly funded by the Australian Department of Health, creating a complete process of “collection via RUM bins in pharmacies—aggregation by pharmaceutical wholesalers—disposal at registered incineration facilities” [4].

To date, substantial empirical evidence has indicated that drug take-back programs represent a safe, eco-friendly, and legally compliant approach to managing medication waste disposal [8,11,21,22,24]. With the in-depth and widespread promotion of drug take-back programs, however, cumulative challenges and gaps have emerged in optimizing the management and implementation of unwanted drug disposal processes with effectiveness, feasibility, and sustainability. Academic research addressing these challenges and gaps is therefore critical to supporting the harmonious operation and maintenance of drug take-back systems [21,25,26,27]. Despite extensive research aimed at advancing theoretical frameworks and addressing practical challenges for optimizing program management and implementation, a systematic mapping of global trends, collaborations, and research hotspots in this field remains absent. In this study, we focused on global academic research on drug take-back programs published over the past 20 years. A bibliographic analysis of the included studies was conducted to provide a detailed overview of research progress in the field of drug take-back programs, thereby identifying research priorities and directing future research efforts.

## 2. Methods

### 2.1. Data Sources and Search Strategy

All academic studies included in the present study were retrieved from the Web of Science (WoS)™ Core Database, an authoritative digital literature retrieval platform provided by Clarivate (version ^©^ 2021 Clarivate). The search query was: TS = (“take-bake” OR “collection system” OR “return scheme” OR “returned”) AND AB = (“unused medicine(s)” OR “unwanted medicine(s)” OR “expired medicine(s)” OR “unused drug(s)” OR “unwanted drug(s)” OR “expired drug(s)” OR “unused pharmaceutical(s)” OR “unwanted pharmaceutical(s)” OR “expired pharmaceutical(s)” OR “unused medication(s)” OR “unwanted medication(s)” OR “expired medication(s)”). Data retrieval covered the period from 2005 to 2025, initially identifying 328 records across eight article types. Among these, 172 were research articles, accounting for 52.44% of all records. The remaining seven article types included Review Article (*n* = 46), Early Access (*n* = 35), Letter (*n* = 27), Editorial (*n* = 25), Proceeding Paper (*n* = 13), Book Chapters (*n* = 6), and Correction (*n* = 4). The inclusion criteria were peer-reviewed research articles written in English, focusing on drug take-back programs, published between 2005 and 2025 in academic journals indexed in the WoS. The exclusion criteria included: (1) non-research article types; (2) publications lacking in-depth analysis of the core research questions related to drug take-back programs; (3) studies addressing the take-back of waste products unrelated to drugs, such as medical sharps, medical product packaging, single-use or disposable insulin pens, and other medical devices. All identified articles were manually reviewed and verified by two independent researchers, and a total of 149 eligible records were finally included in this study. 

### 2.2. Analysis Tools

**Figure 1:**
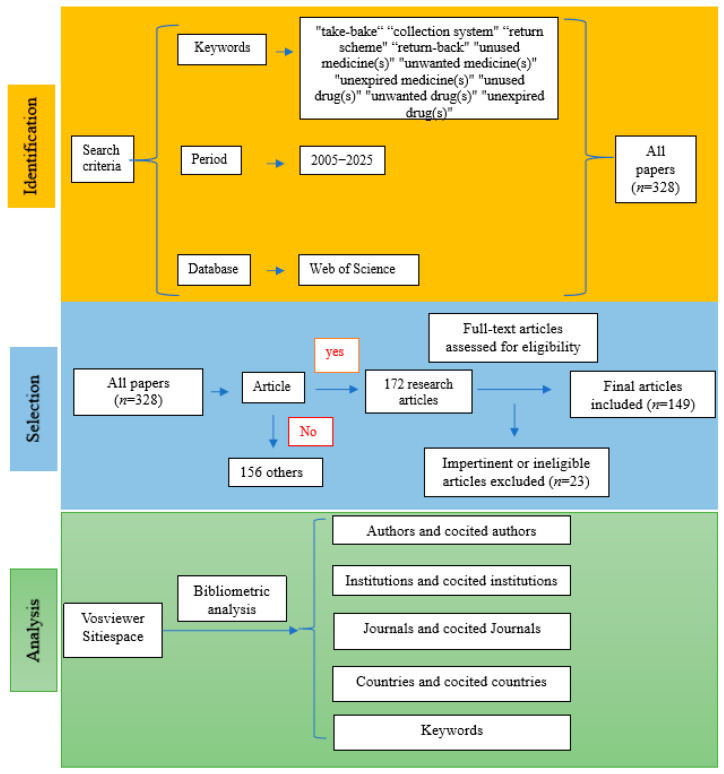
Flowchart of the literature screening process and research framework.

All 149 included records were exported as plain text files, containing complete bibliographic information for each record and their respective cited references. Microsoft Office Excel 2019, VOSviewer, and CiteSpace were used for the analysis of the metadata derived from these records. Microsoft Office Excel 2019 was employed to analyze publication counts, citation data, and the H-indexes of the included studies. Analysis of publication trends was conducted using an exponential growth model in Excel, with the formula specified as: $R^{2}=1-\frac{\sum {\left(y_{i}-{\hat{y}}_{i}\right)}^{2}}{\sum {\left(y_{i}-\bar{y}\right)}^{2}}$. VOSviewer (version 1.6.17), a Java-based bibliometric software, was used to explore collaborative networks of authors, institutions, and countries, as well as to visualize the keyword co-occurrence network. The software CiteSpace (version 6.1.R3 Advanced) was utilized for additional visualization analyses. In addition, narrative data extracted from each included article were analyzed to identify the core research themes. The literature screening process and research framework of the present study are illustrated in Figure 1.

## 3. Results

### 3.1. Publication and Citation Trends

**Figure 2:**
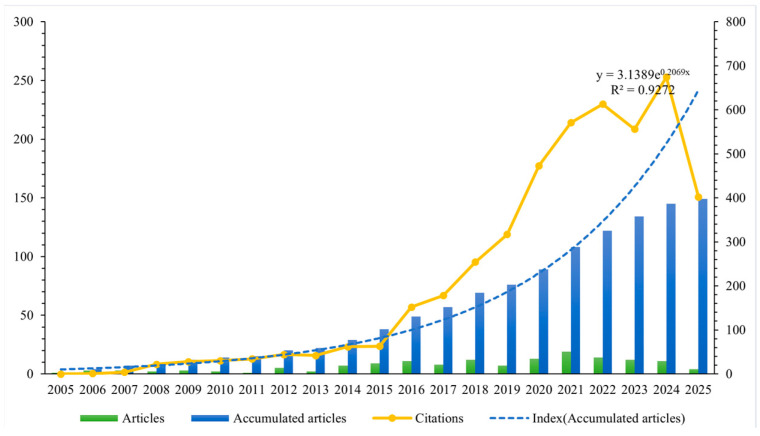
Number of cumulative publications and citations per year. Blue bars represent the cumulative number of publications per year; the blue dotted line depicts the exponential growth function fitted to the cumulative publication data; the orange solid line represents the annual citation counts of the included publications.

The annual distribution of literature quantity is presented in Figure 2. The results indicated that, although only one relevant article was published in 2005 [28] and three in 2006 [29,30,31], international academic interest in drug take-back programs has gradually increased over the past 20 years. Notably, the number of published research articles rose from seven in 2014, reaching a peak annual publication volume of 19 in 2021. An exponential growth function was applied to all included articles to assess the relationship between publication year and cumulative number of publications, showing a strong correlation with an R^2^ of 0.9272. In addition, Figure 2 also reveals a significant upward trend in the annual citation counts of the included publications.

### 3.2. Country Performance

A total of 52 countries/regions across all continents, excluding Antarctica, contributed to the included publications. The top 10 countries/regions ranked by publication output are presented in Table 1. Results showed that the included studies originated from geographically diverse countries/regions, reflecting broad global interest and research engagement in the field of drug take-back programs. The US emerged as the most significant contributor, with 54 publications accounting for 36.24% of all included articles, followed by China (11 articles, 7.38%), England (9 articles, 6.04%), and Brazil (8 articles, 5.37%). Collectively, the top 10 countries contributed to 114 studies on drug take-back programs, representing 76.51% of all included publications. Both developed and developing countries have contributed to advancing scientific research in this field. Moreover, the US also held absolute global leadership in terms of total citation frequency, with citations reaching up to 1407 times; while each of the other countries accumulated only a few hundred citations over the past 20 years, suggesting that studies originating from the US have exerted the highest influence on research related to drug take-back programs.

**Figure 3:**
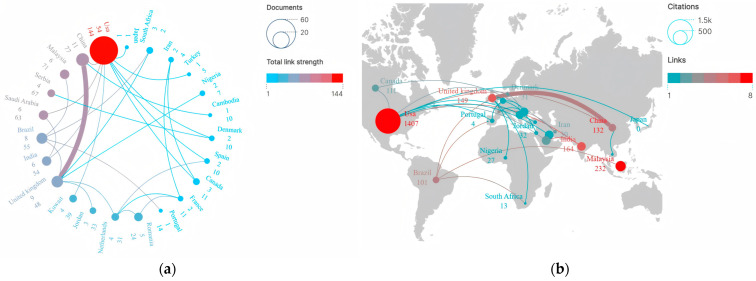
Country performance and academic collaboration in drug take-back research. (**a**) Publication volume and collaboration network. Nodes in the graph represent individual countries/regions, the sizes of nodes correspond to the total research output of a given country/region in international collaborations, and line thickness indicates the strength and frequency of collaboration between connected countries/regions. (**b**) Citation frequency and cooperation intensity. Node size corresponds to citation counts. Node color represents the frequency of citation collaboration for each country/region, with colors ranging from blue to red indicating increasing citation collaboration frequency.

Figure 3a illustrates the collaboration network among countries/regions in scientific research on drug take-back programs. Results showed that over the past 20 years, a total of 23 countries have engaged in active transnational collaborations in this field. Among these, the US played a central role in collaborative research, establishing extensive academic partnerships with China, Saudi Arabia, India, Kuwait, Canada, Turkey, and other countries, forming a collaboration network with 9 interconnecting lines. However, the thickness of the lines connecting countries/regions indicated that the intensity of publication cooperation remained relatively weak. Figure 3b displays the inter-country citation distribution via a world map. Among these 23 countries, the top 5 with the highest inter-country citation frequencies were the US (1407 citations), Malaysia (232 citations), England (179 citations), Saudi Arabia (179 citations), and India (164 citations). Results showed that transnational citations have generally been infrequent over the past 20 years. The US (8), Malaysia (7), India (6), and the United Kingdom (6) tended to cite relevant research from each other, while China (4) had limited collaborative engagement with other countries in drug take-back research.

### 3.3. Institution Performance

Over the past 20 years, a total of 310 institutions worldwide have contributed to academic research on drug take-back programs. The top 10 influential institutions, ranked by publication count, are presented in Table 2. Mercer University (US) topped the list with 5 published articles, followed by the University of North Carolina (US) and King Saud University (Saudi Arabia), each contributing 4 articles.

**Figure 4:**
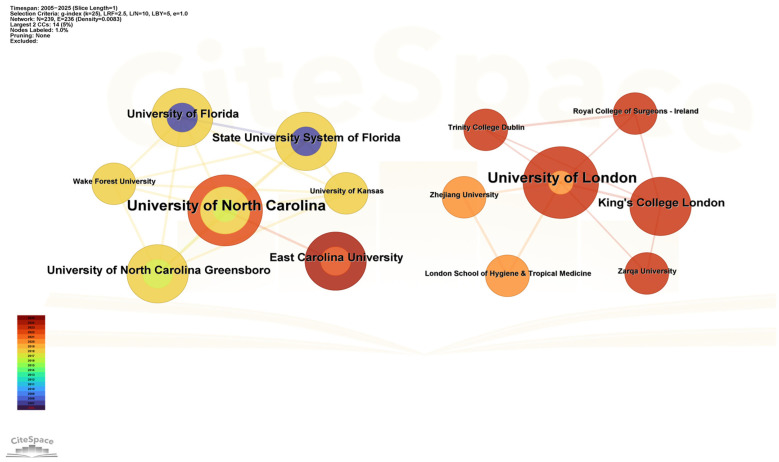
Collaborative network of national institutions. Nodes in the graph represent individual institutions, node size corresponds to the number of articles published by each institution, and the thickness of connecting lines reflects the strength of the collaborative relationship between paired institutions. The color and thickness of a node’s inner circle indicate the number of articles published by the institution in different years.

Figure 4 presents a co-occurrence network of institutional collaborations. Results showed that over the past 20 years, two academic institutions, the University of North Carolina and the University of London, have each formed collaboration networks centered around themselves. The University of North Carolina emerged as the most influential institution engaged in drug take-back research within the US, with the highest number of collaborative connections to institutions including East Carolina University, the University of North Carolina Greensboro, and Wake Forest University. The University of London, meanwhile, maintained close collaborations not only with European institutions (e.g., the London School of Hygiene and Tropical Medicine, King’s College London, and Trinity College Dublin) but also with Asian universities such as Zhejiang University (China) and Zarqa University (Jordan), suggesting a pivotal role of the University of London in fostering intercontinental research collaborations between Europe and Asia. These data indicate that cross-institutional collaborations led by top institutions serve as a crucial driving force for advancing research in the field of drug take-back programs.

### 3.4. Author Performance

A total of 619 authors contributed to the included articles. Findings indicated that the vast majority of these authors (*n* = 564, 91.11%) contributed only a single publication over the past 20 years. Table 3 presents the top 10 most productive authors, with Professor Banga Ajay K., Fowler William, and Andrew Korey, all from the US, topping the list with 5 articles each. Among them, Banga Ajay K. had the strongest academic impact, accumulating 30 total citations and a total link strength of 13. Notably, 60% of the top 10 most active authors were from the US.

**Figure 5:**
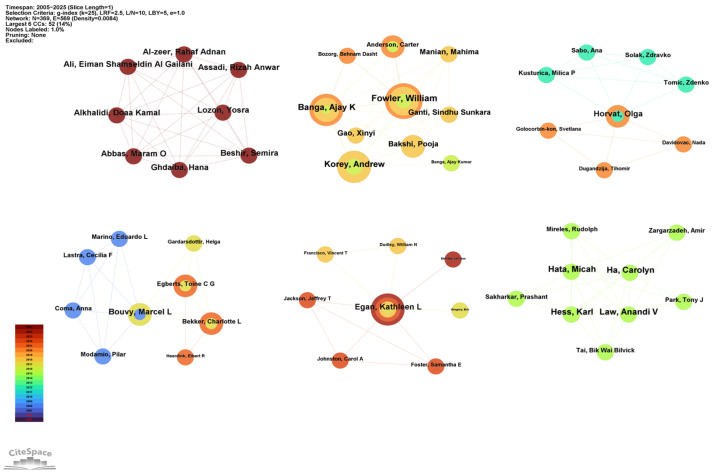
Collaborative network of authors. Nodes in the graph represent individual authors. Node size corresponds to the number of publications by each author, and node links indicate collaborative relationships between authors. The color and thickness of a node’s inner circle represent the number of articles the author published in different years.

The authors’ partnership network is presented in Figure 5. Co-authorship analysis identified six distinct clusters, suggesting strong teamwork dynamics within the field of drug take-back research. The core authors of these six clusters were as follows: Alkhalidi Doaa Kamal (Dubai, total link = 7), Fowler William (US, total link = 15), Bouvy Marcel L (Netherlands, total link = 6), Egan Kathleen L (US, total link = 7), Horvat Olga (Serbia, total link = 8), and Ha Carolyn (Canada, total link = 8). All these core authors collaborated closely with other authors from their respective institutions, highlighting strong intra-institutional ties.

### 3.5. Distribution of Journals and Disciplines

**Figure 6:**
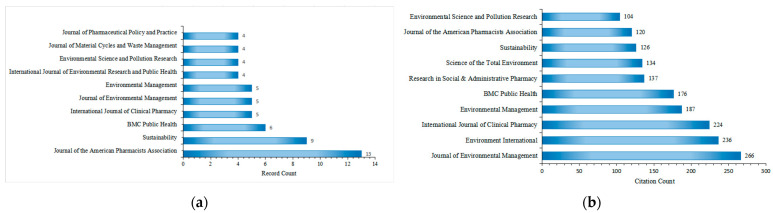
Top 10 journals by publication count (**a**) and citation count (**b**) in the field of drug take-back research.

The 149 included articles related to drug take-back programs were published in 95 scholarly journals, with 71 of these journals publishing only one article in this field over the past 20 years. Figure 6a lists the top 10 journals by publication count. *The Journal of the American Pharmacists Association* ranked first with 13 publications, followed by *Sustainability* (9 publications), *BMC Public Health* (5 publications), *International Journal of Clinical Pharmacy* (5 publications), and *Journal of Environmental Management* (5 publications). All top 10 journals have an Impact Factor (IF) exceeding 2.5, with *Environment International* having the highest IF at 10.3. During the study period (2005–2025), the 149 included articles were cited approximately 4520 times by 2059 citing articles, with an average of 30.13 citations per article. The top 10 journals by citation count are presented in Figure 6b. Research on drug take-back published in the *Journal of Environmental Management* received the most citations (266), followed by *Environment International* (236 citations) and the *International Journal of Clinical Pharmacy* (224 citations). The most cited individual article in this field was published in *Environment International* [32].

**Figure 7:**
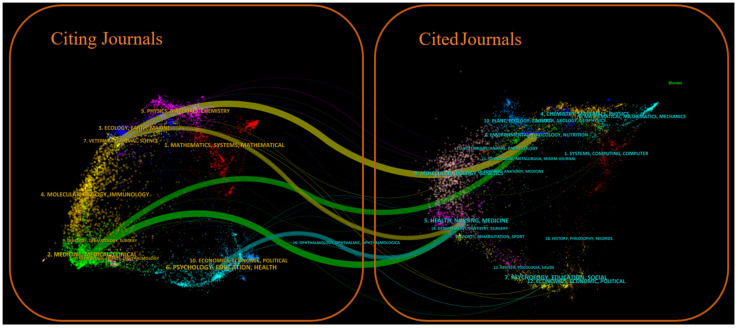
Dual-map overlay of citing and cited journals for drug take-back research. The left panel of the graph illustrates the distribution of citing journals, and the right panel shows the journals being cited. Labels in the panels indicate the disciplines covered by these journals, while colored pathways demonstrate the citation flow from the left-side citing literature to the right-side cited literature.

**Figure 8:**
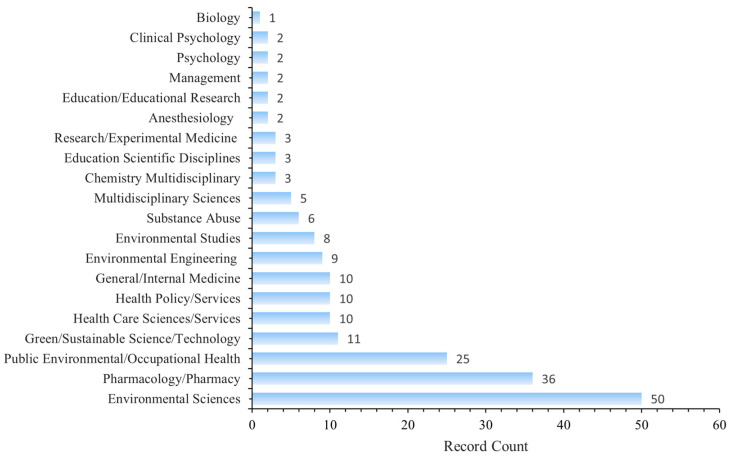
Top 20 categories of drug take-back research.

Figure 7 shows a dual-map overlay of citing and cited journals, identifying four primary knowledge diffusion directions across disciplines. Overall, most articles published in journals focused on Environmental/Toxicology/Nutrition/Health/Nursing/Medicine were frequently cited by journals in Physics/Materials/Chemistry/Mathematics/Systems/Mathematical Sciences. In comparison, articles from Molecular/Biology/Genetics journals were often cited by journals categorized under Medicine/Medical/Clinical Research. To characterize the discipline distribution of drug take-back research, we categorized all included publications by their research fields. Subject categories were assigned by WoS based on the journal in which each article was published. Articles assigned to multiple WoS categories were counted once per category. Results showed that over the past 20 years, journals publishing drug take-back-related articles have covered 36 WoS categories. Of the 149 included articles, 55 (36.91%) belong to multiple categories: 36 fall into two categories, 17 into three categories, 1 into four categories, and 1 into five categories. These findings highlight the interdisciplinary nature of drug take-back research. The top 20 categories are presented in Figure 8. Environmental Sciences ranked first with 50 publications (33.55%), followed by “Pharmacology/Pharmacy” (24.16%), and “Public Environmental/Occupational Health” (16.78%).

### 3.6. Cited References

To identify the knowledge foundation of the drug take-back research field, we analyzed the cited references of the included articles. Table 4 presents the top 10 references with the highest citation frequencies, representing the most influential foundational works. The most highly cited article, “*Beyond the medicine cabinet: An analysis of where and why medications accumulate*” by Ruhoy and Daughton [32], has accumulated 185 citations to date. This study comprehensively analyzed the factors contributing to the generation of unwanted drugs and provided an overview of locations and sites in society where medications accumulate and require disposal. Importantly, the authors highlighted the critical role of drug take-back/return programs in mitigating pollution from improper medication disposal, thereby establishing a foundational framework for subsequent research in this field. The second most-cited article, published by Law et al. in 2015 [33], has 177 citations. This cross-sectional observational survey examined the extent, type, and cost of unused drugs in US households, as well as the reasons for nonuse. A subset of the survey was conducted at planned drug take-back events hosted by community pharmacies. Results indicated that pharmacies could be recommended as safe take-back sites for unused drugs, and combining drug therapy management with waste disposal education was proposed in this study as an efficient way to improve household adherence with proper disposal practices and reduce the costs associated with unused medication disposal. 

**Figure 9:**
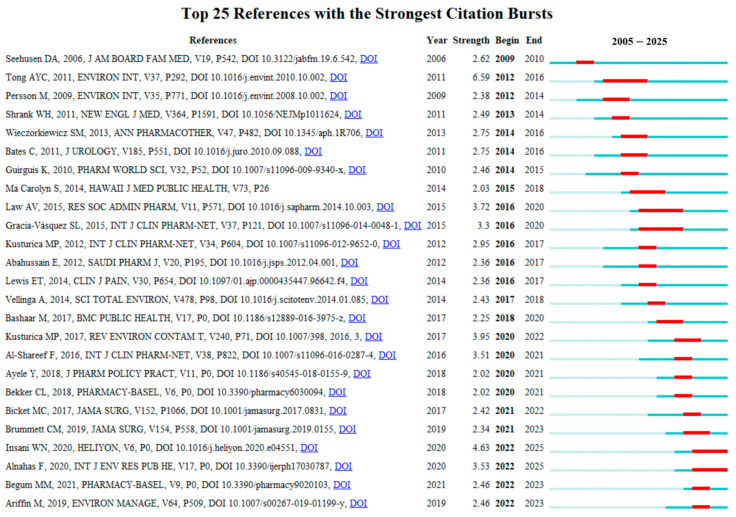
Top 25 references with the strongest citation bursts. Red bars indicate the start and end times of citation bursts, as well as the burst duration.

Figure 9 presents the top 25 references with the strongest citation bursts from 2005 to 2025. These publications are considered to have exerted the most significant impacts on the current research landscape of drug take-back programs. Among them, the reference with the highest citation burst intensity was the article “*Disposal practices for unused medications around the world*” published in *Environment International* by Tong AY in 2011 [10], with a burst score of 6.59. This review synthesized findings from studies on the attitudes and practices of patients and other stakeholders toward medication disposal methods. The authors noted that many countries currently lack standardized medication disposal protocols, and patients have limited awareness of the environmental risks posed by pharmaceuticals, highlighting the need for formalized protocols and public education to support the implementation of drug take-back programs. Notably, prior to 2018, the strongest citation bursts of references primarily focused on surveys of patients’ practices and perceptions regarding drug disposal. In recent years, however, the focus has expanded to other stakeholders (e.g., community pharmacies) [40], consumer willingness to pay for drug take-back programs [41], and additional societal, regulatory, and ethical aspects associated with the implementation of drug take-back systems [42].

### 3.7. Analysis of Author Keywords

**Figure 10:**
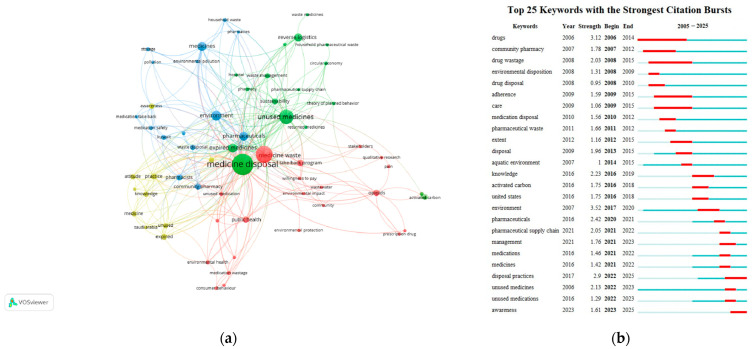
Analysis of keywords. (**a**) Network visualization of the author keywords. Nodes represent individual keywords, and links between nodes indicate co-occurrence relationships between keywords. Different colors correspond to different keyword clusters. (**b**) Top 25 keywords with the strongest citation bursts. Red bars indicate the start and end times of citation bursts, as well as the burst duration.

A total of 64 author keywords were identified in the included articles on drug take-back programs, with each keyword appearing at least twice. “Medicine disposal” had the highest frequency (*n* = 43), followed by “medicine waste” (*n* = 29) and “unused medicines” (*n* = 22). The top 10 most frequent author keywords are detailed in Table 5. Additionally, we analyzed the top 25 author keywords with the strongest citation bursts in this field since 2005. A word cloud generated using VOSviewer from the included articles highlighted core keywords such as “medicine disposal”, “medicine waste”, and “unused medicine”, along with their co-occurrences with terms like “take-back program”, “waste management”, and “community pharmacy” (Figure 10a). Using VOSviewer, the keywords were automatically grouped into four distinct clusters, each represented by a unique color and corresponding to a core research direction or popular topic in drug take-back research: (1) Cluster 1 (yellow), focuses on knowledge, attitudes, and practice (KAP) related to the storage and take-back of unused/expired drugs; (2) Cluster 2 (red), explores the role of drug take-back programs in mitigating environmental and public health hazards caused by medicine waste; (3) Cluster 3 (green), examines unused/expired medicines and sustainable waste management within drug take-back programs; (4) Cluster 4 (blue), centers on the role of pharmacists/pharmacy in drug take-back programs, particularly in guiding households toward proper drug disposal to protect environmental and public health. The strongest link strength of the keywords was observed between “medicine disposal” and “unused medicines”.

As shown in Figure 10b, the keyword “environment” exhibited the strongest historical citation burst strength (3.52), followed by “drugs” (3.12), “disposal practices” (2.9), “pharmaceuticals” (2.42), “knowledge” (2.23), “unused medicines” (2.13), “pharmaceutical supply chain” (2.05), “drug wastage” (2.03), “disposal” (1.96), “community pharmacy” (1.78), and “management” (1.76). Moreover, keywords including “environment”, “pharmaceutical supply chain”, “management”, “disposal practices”, and “awareness” have experienced a surge in usage in recent years, suggesting the current research frontiers and potential future directions for drug take-back research.

**Figure 11:**
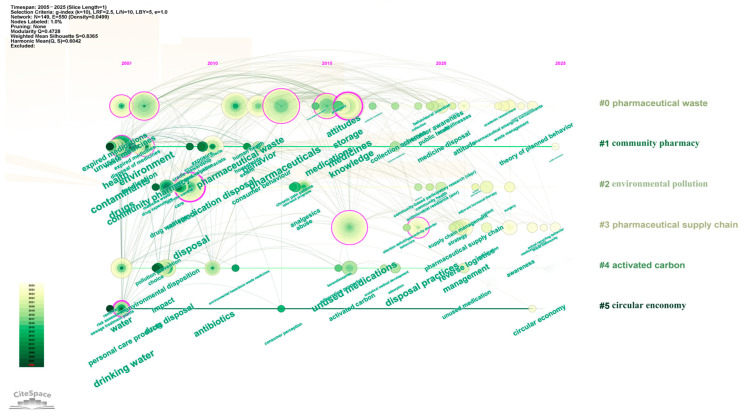
Timeline visualization of drug take-back keywords. Cluster names are displayed in sequential order on the right side of the visualization, and keywords on the same horizontal line belong to the cluster corresponding to that line. Each keyword is represented by a node along the horizontal timeline; the size of each node corresponds to the number of associated keywords, and the horizontal position of each node indicates the year when the keyword first appeared in the included literature.

To clarify the temporal distribution of keyword clusters, Citespace software was further used for keyword analysis. Figure 11 shows that Cluster #0 (pharmaceutical waste) and Cluster #2 (environmental pollution) emerged earliest and have the longest duration, signifying that the environmental benefits associated with drug take-back programs have long been a research hotspot. From 2015 to 2020, Cluster #4 (activated carbon) received relatively greater attention, reflecting increased exploration of harmless treatment technologies for returned pharmaceuticals. After 2020, Cluster #3 (pharmaceutical supply chain) and Cluster #5 (circular economy) became active, suggesting that drug take-back programs are now more frequently examined from the perspective of sustainable development in the pharmaceutical industry.

## 4. Discussion

### 4.1. General Information

This study conducted a bibliometric analysis of global research on drug take-back programs spanning 2005 to 2025. Previous review papers had performed a comprehensive analysis of environmental treatment techniques, including microbial bioremediation [43] and end-of-life destination [44] for pharmaceutical waste management. Nevertheless, these previous reviews mainly focused on the specific techniques used to degrade pharmaceutical pollutants based on analysis of narrative data. To the best of our knowledge, this is the first study to systematically summarize drug take-back research, covering the education, collection, deactivation, reuse, and reverse logistics systems for unwanted pharmaceuticals, from a perspective of drug administration, using a bibliometric method.

Results show a substantial growth in the number of related articles over this period, with a notable surge beginning in 2014. Concurrently, the annual citation counts of the included publications also trended upward, indicating that academic research in this field has received steadily increasing attention over the past 10 years. This timing aligns with the official enforcement of the US federal drug disposal statute in 2014 [3], suggesting the policy may have acted as a key driver for heightened research interest in drug take-back programs.

From a geographical perspective, the global landscape of drug take-back research is characterized by US leadership, with its publication output, total citation frequency, and network centrality being significantly preeminent, revealing a clear geographic concentration of research. These findings are consistent with the strong incentives for drug disposal initiatives, and research at both the national and state levels in the US [3] might identify a research gap regarding the underrepresentation of diverse socioeconomic contexts. Notably, the international collaborative pattern has not yet acted as a primary mode of academic research in the field of drug take-back programs. Such significant disparities highlight pronounced regional inequalities in resource allocation and collaborative opportunities within the drug take-back research system, which may compromise the quality and outcomes of related studies. Potential reasons behind limited international collaboration in this field might lie in constraints imposed by local policies, regulations, and financial support on the implementation of take-back programs [21]. Currently, drug take-back systems vary widely across countries and regions worldwide. Critical details such as collection point locations, practical handover process for unwanted medications, and funding mechanisms differ substantially. Moreover, cross-country/regional cultural and social disparities might influence public awareness, attitudes, and behaviors toward drug take-back systems, leading to highly heterogeneous compliance levels [21]. As early as 2008, the first call was issued for the harmonization of drug take-back systems across the EU to enhance their effectiveness, with a proposal to standardize the information provided to the public [21]. Given that improper drug disposal has become a global concern, there is an urgent need to strengthen international collaboration and establish global research platforms to support in-depth studies on drug take-back programs and address the current fragmentation of systems and research. Therefore, the current leadership of the US in drug take-back research underscores its pivotal role in facilitating international synergistic innovation in this field moving forward.

Consistent with observations on country-level performances, analyses of institutional and author contributions also emphasize that international and cross-institutional collaboration in drug take-back research still requires further strengthening to drive innovation and advance scientific progress in this field. All core authors in the field collaborated closely with other authors from their own institutions, reflecting strong intra-institutional ties. This dominance of intra-institutional collaboration at the author level may be attributed to the lack of objective conditions supporting cross-boundary academic exchanges and scholars’ limited subjective willingness to engage in cross-institutional cooperation, which could lead to fragmented research progress and hinder the integration of scientific advancements across the field. Therefore, researchers working on drug take-back programs should proactively pursue academic collaboration beyond their own institutions, and there is an urgent need to establish an academic exchange platform dedicated to drug take-back research.

The development of strategies for controlling and managing pharmaceuticals as emerging contaminants relies on extensive interdisciplinary research [45,46]. It has been widely accepted that pharmaceutical waste disposal requires a working group of interdisciplinary stakeholders, including those from supply chains, clinical practice, nursing, environmental services, and finance [47]. Our findings indicate that, over the past 20 years, research on drug take-back programs has been primarily dominated by the disciplines of Environmental Science and Pharmacology/Pharmacy, with Environmental Science being markedly predominant. This predominance reflects ongoing concerns among environmental experts about the adverse impacts posed by improper disposal of returned drugs. However, as upstream stakeholders in the implementation of drug take-back programs [48,49], researchers in pharmacy, health sciences, nursing, and medicine should increase their engagement in this field. Furthermore, drug take-back research has emphasized interdisciplinary integration of pharmacy, health sciences, nursing, medicine, environmental science, public environmental/occupational health, green and sustainable science/technology, biotechnology, health policy services, and education, to comprehensively address the environmental and health risks of improper unwanted drug disposal [50,51,52]. Strengthening interdisciplinary exchanges and collaborations in this field could provide innovative technical approaches and research perspectives to support the development of theoretical frameworks and practical models for drug take-back systems.

### 4.2. Main Research Themes

Analysis of keyword clusters summarized the five core themes that have dominated drug take-back research over the past 20 years.

#### 4.2.1. Assessment of KAP Levels Associated with Drug Take-Back and Their Influential Factors

Among the 149 included studies, an overwhelming majority (76 articles, 51.01%) focused on assessing KAP levels related to drug take-back and their potential influential factors among diverse stakeholders, including patients, the general public, healthcare students, university students, pharmacies and pharmacists, pharmaceutical manufacturers and warehouses, and government officials across different regions globally.

To understand actual KAP levels and identify factors that promote drug take-back intention and behavior, KAP-related studies over the past 20 years have covered respondents across 32 countries/regions, including the US [24,33,34,37,53,54,55,56,57,58,59,60,61,62,63,64], China [65,66,67,68,69,70], Saudi Arabia [71,72,73,74], Malaysia [23,75,76,77], Serbia [41,78,79,80], Brazil [81,82,83,84], the United Kingdoms [28,85], India [47,86,87], Kuwait [29,88,89], Australia [4,90], Nigeria [91,92], Jordan [93,94], Romania [38,95], Germany [96], Sweden [31,97], Ireland [36], Indonesia [39], Thailand [98], Poland [99], Tanzania [100], Nepal [101], South Africa [102], Ethiopia [103], Cyprus [104], Algeria [105], Afghanistan [35], Ghana [106], the United Arab Emirates [107], Palestine [108], Croatia [109], Pakistan [110], and Eritrea [111]. As summarized in Supplementary Material Table S1, findings consistently highlight the importance of improving KAP levels related to drug take-back. However, the global drug take-back practice remains generally insufficient. Moreover, knowledge and attitudes are key predictors of individuals’ willingness to participate in/pay for drug take-back programs, as well as their actual engagement. Previous studies collectively revealed the presence of a significant knowledge gap in this area, emphasizing the urgency of awareness-raising initiatives. In addition, recent studies based on the Theory of Planned Behavior have identified the predictive role of subjective norms and perceived behavioral control in drug take-back intention [54,69,75,82,86]. Specifically, subjective norms, along with altruistic values and feelings of responsibility, which stem from surrounding perception or social pressure, can be internalized into personal norms. This process shapes positive attitudes, ultimately exerting a significant impact on sustainable drug take-back intention and behavior [86].

Beyond the single-country KAP surveys mentioned above, Doerr-MacEwen and Haight [30] conducted a study that recruited 27 expert stakeholders representing academia, government, the pharmaceutical industry, and consulting sectors from Canada, the US, and Europe. This study evaluated the experts’ perspectives on precautionary management actions to control pharmaceutical discharge into the environment. Results indicated that, in the interviewees’ view, drug take-back programs combined with public education formed one of the most effective and feasible strategies to reduce the adverse environmental impacts of pharmaceuticals. Another cross-sectional survey targeted 277 pharmacists or pharmacy technicians from six Gulf Cooperation Council countries, assessed the respondents’ practical activities associated with medication waste disposal, and evaluated the importance of these activities in minimizing medication waste [112].

Notably, a certain proportion of the included KAP studies highlight the role of pharmacy/pharmacists in drug take-back programs [28,31,62,63,64,71,74,77,80,83,84,87,89,106,107,108,109,110,112]. For implementing such programs, multiple convenient drop-off locations are provided to enable households to safely dispose of unwanted medications, including pharmacies, clinics, and other healthcare facilities, and community sites [56]. Consumers have expressed a clear preference for pharmacy-based drug take-back services [28,31,62,63,106], compared to other options, such as returning medications to general practice surgeries and using at-home drug disposal products (e.g., deactivation system or mailed envelope). Interviews with pharmacists working in retail pharmacies also showed that they agreed the patient benefits from take-back boxes and acknowledged their own responsibility in drug take-back practice [64]. However, despite consumers’ willingness and pharmacists’ recognition, the practical implementation of pharmacy-based interventions is underwhelming. Egan et al. [64] examined retail pharmacies in Pitt County, North Carolina, US, and found that only 32.3% had drug take-back boxes. Another cross-sectional study, which included the entire population of state pharmacies at the primary healthcare level in Serbia, reported that 23.5% of the studied pharmacies did not collect household pharmaceutical waste at all [80]. Similarly, in Zagreb, Croatia, the volume of medications collected by local pharmacies was below the European average [109]. These findings suggest significant room to strengthen pharmacies’ role in drug take-back implementation. Complex factors might influence the functioning of pharmacy-based drug take-back practices, including pharmacy ownership type, the cost of sustaining the service, distribution of pharmacies, management policies, and the lack of anonymity for disposing of unused drugs [64,80,109].

The active engagement of pharmacists is critical to the success of drug take-back programs. As medication experts and the most accessible healthcare providers, pharmacists play a pivotal role in promoting safe disposal of medications among the public. Nine cross-sectional studies specifically examined pharmacists’ perceptions and practices related to drug take-back [74,77,83,84,87,89,107,108,112], while two additional studies [71,110] focused on the group of pharmacy students as future pharmacists. Collectively, survey results indicate that pharmacists generally recognize the importance of safe medication disposal and their responsibility to guide the public on proper disposal of unwanted medications, and they also hold positive attitudes toward drug take-back programs. However, a gap exists between pharmacists’ beliefs and their actual take-back practices, which may be attributed to insufficient resources and a lack of systemic support.

#### 4.2.2. Treatment of Medications Collected in Drug Take-Back Programs

A key challenge for the effective implementation of drug take-back programs is how to handle returned medications in a safe and eco-friendly manner [101,113]. In traditional drug take-back programs, returned medications are typically destroyed via incineration, a method that can generate toxic air emissions [4]. Our analysis shows the second focus of drug take-back research is exploring or evaluating treatment strategies for medications collected through drug take-back programs, with 37 recent articles addressing this topic. Among them, 15 papers focused on deactivation treatment of unwanted/returned drugs as medical waste [114,115,116,117,118,119,120,121,122,123,124,125,126,127,128], while 22 articles investigated issues related to medicines reuse and reverse logistics systems in the context of drug take-back [8,105,129,130,131,132,133,134,135,136,137,138,139,140,141,142,143,144,145,146,147,148].

##### Deactivation Systems for Returned Medications

Over the past 20 years, five studies have focused on evaluating activated carbon-based deactivation systems for safe disposal of medications, highlighting their role in supporting the full execution of drug take-back programs [114,115,116,117,118]. Deactivation systems are reaction-based setups designed to neutralize the active pharmaceutical ingredients in unused or expired drugs. They work through chemical or physical reactions with deactivation agents, such as activated carbon [114,115,116,117,118], thereby rendering pharmaceutical ingredients biologically inactive and ensuring that if the treated waste later enters the environment, the unused or expired drugs no longer pose ecological risks. All the studies [114,115,116,117,118] examined the Deterra^®^ drug deactivation pouch, a commercial drug deactivation system developed by Verde Environmental Technologies Inc. (Minnetonka, MN, USA). The Deterra^®^ system is a resealable plastic pouch containing granulated activated carbon enclosed in a water-soluble film, and it operates based on MAT12^®^ Molecular Adsorption Technology. Users can perform harmless treatment of unwanted medications via four simple steps: placing medications in the pouch, adding warm tap water, sealing the pouch, and discarding it with household trash. Herwadkar et al. [114] compared the efficiency of four deactivation agents, including an oxidizing agent (sodium percarbonate), a hydrolysis agent (sodium carbonate), and two adsorbents (activated carbon and zeolite), in supporting drug deactivation systems. Pharmaceuticals with varying ionic characteristics or water solubility, including ketoprofen, dexamethasone sodium phosphate, metformin hydrochloride, amoxicillin trihydrate, and fentanyl, were targeted in this study [114]. Results showed that sodium percarbonate and sodium carbonate were only effective for deactivating amoxicillin trihydrate. Deactivation efficiency of zeolite was not sufficiently certain, as it depended on zeolite concentration, the contact time between pharmaceuticals and zeolite, and the physicochemical properties of target pharmaceuticals. Only activated carbon achieved effective deactivation of most tested pharmaceuticals across all dosage forms, indicating its potential as a universal pharmaceutical deactivation agent. The high efficiency of Deterra^®^ system in irreversibly deactivating various pharmaceuticals has been well documented, including prescription sedative medications (alprazolam, temazepam, zolpidem [115], diazepam, lorazepam, buprenorphine [116]), psychoactive medications (methylphenidate hydrochloride, loxapine succinate) [117], and highly abused opioid medications (morphine sulfate, methadone hydrochloride, hydromorphone hydrochloride, and meperidine hydrochloride) [118]. Deactivation rates of 94.0–99.9% were achieved after 8 h [116,117,118]. Moreover, desorption studies showed that after the deactivation process, no active pharmaceutical ingredients were released from the activated carbon materials even when large volumes of water or ethanol were used to wash them, suggesting minimal leaching and environmental impact when the contents of the Deterra^®^ system were sent to landfills [115,116,117,118]. Beyond the Deterra^®^ drug deactivation system, other activated carbon-based commercial products for deactivating active pharmaceutical ingredients include Drug Buster^®^, Rx Destroyer^®^, Pill Catcher^®^, and DisposeRx^®^ [149]. As a convenient, safe, effective, and eco-friendly method for unused medication disposal suitable for household and healthcare settings, activated carbon-based systems are acceptable to patients and help improve their compliance with drug take-back programs [119,121,122,123,124,125,126,127,128]. Nevertheless, a multi-arm parallel-group randomized controlled trial revealed that, among individuals who received a short-term opioid prescription from outpatient pharmacies, passive provision of a drug deactivation system did not enhance the disposal rate of leftover opioids compared to no intervention [120], highlighting the critical need for patient and caregiver education, as well as their active engagement, to fully unlock the effectiveness of such deactivation systems.

##### Medicines Reuse and Reverse Logistics Systems

As an alternative take-back strategy, redispensing or donating patient-returned medications, after verifying their quality, to other patients offers an eco-friendly and cost-effective approach to reduce the environmental burden of pharmaceuticals. Mackridge and Marriott [129] analyzed medications returned to eight community pharmacies and five general practice surgeries in Eastern Birmingham, the United Kingdom. A registered pharmacist was employed in this study to professionally assess whether these medications were suitable for reuse. The returned medications had a median of 17 months remaining until expiration, and approximately one-quarter (1248 out of 4291) were deemed suitable for potential reuse. Similarly, a three-year European medication donation program collected 53,644 boxes of returned medicines, of which 43% (23,145 boxes) were qualified for donation. These qualified medicines were then distributed to 14 beneficiary organizations across Europe, Latin America, and Africa [131]. These findings indicate that a significant portion of medications collected through drug take-back programs retains substantial financial value, supporting the feasibility of medication reuse. Five additional studies explored stakeholder perspectives and willingness toward medication reuse [131,132,133,134,135]. Participants included patients, young people, taxpayers, pharmacy students, pharmacists, pharmaceutical supply specialists, pharmaceutical manufacturers, beneficiary organizations involved in medication donation programs, environmentalists, and pharmacy researchers. Results showed that, despite divergent opinions among stakeholders, broad agreement on medication reuse could be reached if key concerns are addressed, safety requirements for redispensing are met, and the reuse process is clearly defined and well managed.

A small number of studies have proposed integrating artificial intelligence (AI)-based classification technologies into drug take-back programs to automatically classify returned medications for proper disposal or reuse [136,138]. AI-based classification, in the context of drug take-back programs, refers to a technology-driven process that leverages AI algorithms and techniques to automatically identify, categorize, and sort returned unused or expired medications [136,138]. A key barrier to medication reuse is the need for quality and safety validation of returned medicines. Hui et al. [136] addressed this by developing a Digital Time Temperature Humidity Indicator (dTTHI) smart sensor with cloud connectivity, which was designed based on Internet of Things (IoT) architecture derived from the Reuse of Medicines through Informatics, Networks and Digital Sensors (ReMINDS) ecosystem. This sensor was integrated onto the pharmaceutical packaging materials, enabling real-time quality and safety monitoring of packaged medicines, remote information sharing with stakeholders via the Internet, and encouraging stakeholders to adopt a behavioral change and return unused medicines in good condition to pharmacies. Thereby, this sensor acts as a technological component to facilitate medicine return and redispensing. Banjar et al. [138] developed another AI-based system, including software composed of a web-based expert system to educate consumers on proper drug disposal, a chatbot for consumer interaction, and a hardware device for collecting and automatically classifying medications into disposal or donation categories. However, these technologies remain in the research stage, and their operational and ethical assessments are entirely lacking to date. Moreover, economic constraints cannot be overlooked for implementing such innovative interventions, especially in low- and middle-income countries. For example, based on online small-quantity purchase prices (approximately 100 units), the estimated production cost of the dTTHI is roughly GBP 10 [136], which would increase the overall cost of medication redispensing. Another interesting study explored the repurposing of leftover hydroxychloroquine sulfate tablets, largely accumulated in households during the COVID-19 pandemic and later returned via drug take-back programs, as corrosion inhibitors and fungicides in oilfield water treatment [139].

Over the past 20 years, scholars have explored conceptual models for reusing or recycling surplus medications before expiration from the perspective of sustainable development. Tat and Heydari [137], as well as Nematollahi and Hosseini-Motlagh [140], proposed developing a novel collaborative model, which involves upstream members (pharma-suppliers) and downstream members (pharma-retailers) in the pharmaceutical supply chain, aiming to collect, donate, and resell unwanted medications in an environmentally responsible manner. By using the Stackelberg game framework [8], an optimal recycling system for unwanted medicines was designed through leveraging the sustainable synergy among customers, governments, pharmaceutical manufacturers, and pharmacies. Li et al. [141] focused on the critical role of the community in resource sharing and recycling, and proposed a surplus medicine community-sharing service system. In particular, eight studies [105,142,143,144,145,146,147,148] have highlighted and investigated pharmaceutical reverse logistics systems and practices, based on the concept of reverse logistics. As a widely adopted waste management strategy in supply chain management, reverse logistics refers to the systematic process of managing the flow of products and materials from the point of consumption (such as end-users, households, or retail outlets) back to their point of origin (like manufacturers, or distributors). This direction of flow stands in contrast to the conventional supply chain, which typically moves goods from producers to consumers [105,142,143,144,145,146,147,148].

#### 4.2.3. Evaluation of Drug Take-Back Intervention Activities

Of importance, 25 additional studies evaluated the potential effectiveness of drug take-back intervention activities by assessing KAP levels among intervention participants and/or measuring the quantity and types of medications collected [11,21,25,26,27,42,120,150,151,152,153,154,155,156,157,158,159,160,161,162,163,164,165,166,167]. At the national level, Kuwait has successfully implemented a national-level drug take-back campaign leveraging a comprehensive national media system and multisectoral collaboration [150]. Aligned with the 2022 World Patient Safety Day theme “*Medication without Harm, a Multisectoral Initiative*”, this campaign effectively boosted public participation in drug take-back practices [150]. Beyond national programs, the acceptability, feasibility, and potential efficacy of statewide or provincial drug take-back initiatives have been documented, including New Jersey’s social marketing campaign “the American Medicine Chest Challenge” [151], Maine’s Safe Medicine Disposal for ME mail-back program run by the Maine Drug Enforcement Administration [27], 11 state-wide Drug Take Back events organized by the Hawai‘i Narcotics Enforcement Division in partnership with a local US university [168], the Expired Medication Program instituted by the Department of Health of the State of Nuevo Leo’n, Mexico [154], and a community-based antibiotic take-back program embedded in a “bartering market” for recyclables in Zhejiang province, China [48]. These initiatives have been shown to promote the disposal of expired or unwanted medications at collection sites. At the local scale, a US example was “Denton Drug Disposal Days” in Denton, Texas, a series of drug take-back events developed through collaboration between local officials and university researchers [25]. Qualitative comparisons between medication collection data from these events and data from the North Texas Poison Center suggested that the initiative may reduce the risk of accidental poisoning or intentional abuse. Moreover, participant surveys via telephone interviews and Geographic Information Systems (GIS) analysis identified a definitive travel threshold for participation in drug take-back events, with most residents preferring “local” programs and a maximum acceptable travel distance of fewer than 5 miles. In Dane County, Wisconsin, MedDrop™ programs are held biannually as drive-through services where volunteers collect unwanted medications from vehicle occupants [155]. Welham et al. [155] analyzed data from a 4-h MedDrop™ take-back event, during which 761 households returned 680 kg of medications for disposal. Another local US initiative is a continuous drug take-back program in Alachua County, Florida, designed as a customer “self-serve” system [160]. Across 12 collection locations, participants deposited unwanted medications directly into containers filled with tap water or an aqueous acidic solution—both of which dissolve the medications and render them unusable. Particularly in densely populated countries and regions, community-centered interventions have been recognized as playing a dominant role in drug take-back practices [11,26,48,156,157,158,159,161,162,163,164,166], with common forms including community education, door-to-door collection of unwanted medicines, distribution of drug deactivation bags, and placement of medication drop boxes. A representative example is a US study conducted between 2011 and 2015, which recruited sites across six states via the Pharmwaste listserv, a “national pharmaceutical waste management” discussion forum [26]. Eighty community events were held in diverse forms, such as drive-up/drop-off and walk-up, on the Drug Enforcement Administration’s National Medication Take Back day, with participants recruited through newspaper advertisements, pharmacy flyers, and word of mouth. Pharmacy and other health professional students, faculty, and community partners were included as event volunteers. Study participation sites were provided no-cost access to the Pharmaceutical Collection Monitoring System™ (PCMS™), a web-based pharmaceutical collection logging tool. Multi-event and multi-state data from this study showed that these community events collected more than 10,270 prescriptions (280,813 units of medication) over the five-year period.

Interestingly, two studies identified a key insight in educational interventions for boosting residents’ drug take-back awareness: Delivering environmental health information through a loss-framing approach, which emphasizes the negative environmental consequences of improper drug disposal, significantly boosts the likelihood of households returning pharmaceutical waste [11,21]. Among European respondents, improper disposal of unwanted drugs was more commonly viewed as an environmental issue rather than a health concern [21]. Similarly, a comparison involving Chinese households showed that those informed about the environmental hazards of pharmaceutical emerging contaminants were more engaged in drug take-back programs than those who received information focused on the health risks of drug misuse or overuse [11]. These findings suggest that eco-directed brief awareness-raising interventions could act as a cost-efficient, convenient, and impactful education strategy to improve public compliance with drug take-back initiatives.

Among the 25 intervention studies, two focused on designing drug take-back programs for specific populations [152,153]. Lewis et al. [152] developed and implemented a novel opiate reclamation initiative to collect unused opiate pain medications in the community. This program targeted general surgery postoperative patients, a group with direct access to opiates, by requiring them to bring unused opiates to their routine postoperative follow-up appointments for disposal in secure drug take-back bins. The other study designed a take-back intervention for unclaimed student medications in a large local school district in the San Diego area, covering 180 elementary, middle, and high schools [153]. During a district-wide nurses’ meeting, an Environmentally Responsible Medication Disposal Protocol was delivered and required the school nurses to send verbal or written reminders to parents, thereby supervising students to return their unwanted drugs to the school nurses. Additionally, a rule was implemented to prohibit students from taking medications home at the end of the school year. This program successfully collected a large quantity of unclaimed medications in one school year, and a follow-up survey on nurses’ attitudes and practices confirmed the program was highly feasible and acceptable as an incentive for safe, environmentally responsible medication disposal.

Healthcare students, including student pharmacists, have long been viewed as valuable force multipliers in community-based health services, owing to their vibrancy, professional knowledge and skills, and understanding of local cultural customs [46]. Over the past 20 years, two published articles have highlighted student pharmacists’ valuable role in implementing community-based drug take-back programs [163,164]. In a study conducted by Gray-Winnett et al. [164], student pharmacists worked cooperatively with community officials and businesses to plan promotional activities for the take-back program and manage the collection of unused medications, thereby supporting the program’s successful implementation. Similarly, Abrons et al. [163] designed a student pharmacist-led educational initiative aiming at improving public awareness of safe drug disposal, which was integrated into a community drug take-back program. Post-education survey results showed that most respondents were willing to switch from their previous medication disposal habits to participating in take-back programs. Additionally, respondents’ awareness of the risks associated with improper medication disposal was significantly increased, and 59.7% of respondents strongly agreed that student pharmacists serve as a reliable resource for public health information on safe medication disposal and drug take-back.

However, among the 25 studies evaluating the effectiveness of drug take-back interventions, a significant proportion only relied on classifying and quantitatively assessing returned medications [26,27,154,155,157,159,161,162,165] and lacked a pre-test–post-test control group design, which might weaken the reliability of their findings. More well-designed, evidence-based intervention studies are still required to accurately verify and explore the effectiveness of drug take-back programs.

#### 4.2.4. Analysis of Regulations and Policies Regarding Medication Waste Management

Among studies about drug take-back programs retrieved from the WoS core database, 7 studies published since 2005 have focused on analyzing regulations and policies related to medication waste management [169,170,171,172,173,174,175]. Bellan et al. [169] conducted a critical analysis of medication waste disposal management models and discussed international experience of drug take-back programs, such as pharmacies in British Columbia participating in the Medications Return Program, European programs for reverse medication logistics, and current Brazilian regulations. Zimmermann et al. [170] proposed establishing a legal framework to allow pharmacies to collect unused, expired, or spoiled narcotics and psychotropic drugs from patients. Three studies from Romania carried out legal documentary research on pharmaceutical waste management-related legislation, highlighting the need to develop a legal framework as part of green pharmacy practices [171,172,175]. Under Romania’s National Law No. 95/2006 on healthcare reform, the National Agency for Medicines and Medical Devices is tasked with assigning pharmacies and drugstores to adequate take-back systems for collecting and disposing of unused/expired drugs, though this obligation is not enforced. From a Ugandan perspective, researchers discussed the necessity of strengthening pharmaceutical supply chain management, along with supporting regulatory mechanisms and accountability systems, to mitigate the health and environmental risks posed by unwanted medications in low- and middle-income countries [173]. Barnett-Itzhaki et al. [174] investigated global approaches to household medical waste collection and disposal, then put forward recommendations for Israel’s drug take-back system, including permitting a diverse range of institutions (e.g., pharmacies, police station drop boxes, hospitals, post offices, and even large supermarkets) to collect household unwanted medications, and legally mandating pharmaceutical manufacturers to fund drug take-back programs.

#### 4.2.5. Role of Drug Take-Back Programs in Mitigating Environmental and Public Health Hazards from Medicine Waste

Since 2005, 4 published articles have focused on the environmental and health benefits of managing medicine waste through take-bake programs [32,176,177,178]. Ruhoy and Daughton [32] analyzed the processes and behaviors associated with medication accumulation and disposal, and identified consumer “take-back” or “return” programs as key opportunities to prevent pharmaceutical pollution and reduce such waste at the source. An interesting study conducted by Stoddard and Huggett [178] monitored the concentration of hydrocodone, a common pharmaceutical contaminant, in wastewater effluent released from a wastewater treatment plant in Denton, Texas, US, before and after the local “Denton Drug Disposal Day” take-back initiative targeting Denton residents. Results showed that this program successfully reduced pharmaceutical emissions into the environment, providing direct evidence of its environmental mitigation effect. Pereira et al. [177] framed drug take-back programs as a potential benchmark for reverse logistics systems handling Household Waste Medicine (HWM). Using the Brazilian HWM situation as an example, they argued that the lack of a national drug take-back program in Brazil has worsened the significant environmental and public health challenges linked to HWMs [177].

However, whether drug take-back systems play an irreplaceable role in mitigating environmental risks associated with pharmaceutical disposal remains controversial. The US FDA recommends that when drug take-back programs are not readily available, expired or unwanted drugs can be flushed down the sink or toilet, which is particularly suitable for prescription drugs with potentially lethal overdose risks. To support this guidance, Khan et al. [176] assessed the potential ecological and health risks associated with the environmental release of 15 active pharmaceutical ingredients on the FDA’s “flush list”, a list of drug products the FDA recommends flushing to dispose of unused quantities. Results showed that, except for a small number of active pharmaceutical ingredients lacking sufficient eco-toxicological data, most studied pharmaceuticals on the list posed negligible eco-toxicological risks after being disposed of via flushing, whether as individual compounds or in mixtures. This finding challenges the idea that take-back systems are the only safe option for pharmaceutical disposal, suggesting flushing can be a low-risk alternative in specific cases, particularly when take-back access is limited.

### 4.3. Future Prospects

Our findings demonstrated that current research on drug take-back programs is mostly concentrated in environmental and pharmacological disciplines, with a heavy focus on the knowledge, attitudes, and practices (KAP) of diverse stakeholders. However, the implementation and effectiveness of drug take-back programs vary substantially across regions, influenced by socioeconomic conditions, regulatory frameworks, and cultural contexts. To date, there is no universally recognized paradigm for drug take-back systems, even in the US, which dominates related research. Meaning landmark work is still needed to develop effective, feasible, and widely applicable programs that can offer actionable recommendations to policymakers.

Moreover, the dominance of data from the US and other high-income countries might distort the comprehensive picture of global drug take-back research. Findings from developed regions cannot be directly applied to low- and middle-income settings, due to constraints like policy gaps, inadequate infrastructure, and cultural barriers [173]. Currently, low- and middle-income countries face major barriers to promoting take-back programs because of resource constraints such as limited personnel, funding, and technology [22]. In China, local governments and pharmaceutical enterprises have launched proactive drug take-back initiatives, but these lack nationwide legal support and a unified funding pool [8,9]. In Indonesia and Malaysia, no official drug take-back policies exist yet, with some pharmacies offering voluntary services driven by social and community responsibility. These services have no fixed funding support or standardized collection infrastructure, leading to relatively weak operational stability [22,23]. In Uganda, many expired pharmaceuticals come from donated stockpiles stored in national stores and public-sector health facilities, which is quite different from the prevalence of household storage of unused medicines in high-income countries. Therefore, for the low- and middle-income countries like Uganda, tailored national-level drug take-back policies are essential, such as establishing channels to redistribute excess pharmaceutical inventory to public and private health facilities at all levels, prioritizing investments in ultra-high-temperature incineration, evaluating and adopting low-cost methods of drug disposal (e.g., waste immobilization or engineered landfill via encapsulation or inertization) [173]. Nevertheless, no research has systematically investigated drug take-back models suited to low- and middle-income settings, accounting for their unique policy, infrastructure, and cultural traits.

In addition, limited international collaboration and interdisciplinary integration highlight a critical gap and a substantial opportunity to strengthen the global response to pharmaceutical waste, which can be achieved through more unified policy frameworks, targeted public engagement strategies, and innovative technological solutions. Future efforts should prioritize cross-sectoral cooperation and evidence-based interventions to optimize the safety, sustainability, and accessibility of drug take-back systems worldwide. However, multiple real-world limitations, such as political and regulatory differences, variations in healthcare infrastructure, and divergent cultural contexts, might undermine the utility of academic findings or models for policymakers in diverse regions. Therefore, exploring how to design and adapt tailored drug take-back measures to local conditions should be a key focus of future research.

### 4.4. Limitations

There were several limitations in the present study. First, the bibliometric approach employed in this study provides breadth in covering drug take-back program research but lacks depth of insight. Second, the study relies solely on data from the WoS database, excludes regional studies, non-English or gray literature, which are critical for capturing diverse research outputs globally. This omission hinders a comprehensive understanding of the global diversity in policies, stakeholder perspectives, and on-the-ground implementation challenges related to drug take-back initiatives. In addition, many published articles use alternative terms to describe drug take-back programs, such as “collection system for unused/expired/unwanted pharmaceuticals/medications” [105,160] and “medicine return” [66]. The broad and inconsistent search terms used in this study may have led to the exclusion of relevant literature, skewing results toward countries and research fields with better database indexing and more English-language outputs. Moreover, the study mainly relied on descriptive analysis of bibliometric data. The correlations reported in statistical modeling (e.g., exponential growth function) lack controls for confounding variables and fail to account for publication bias or research clustering. To validate the robustness of our findings, further inferential analyses and more rigorous statistical considerations are required.

## 5. Conclusions

In summary, this bibliometric review systematically mapped and synthesized global academic research on drug take-back programs over the past two decades. Our analysis elucidated publication trends, collaborative networks, disciplinary distributions, and emerging research themes, providing a comprehensive overview of the current state and future directions of this field.

## Figures and Tables

**Table 1:** Top 10 countries by publication output.

Rank	Country	Publication Count	% of the Included Articles	Average per Item	H-Index	Citations	Total Link Strength *
1	US	54	36.24	32.38	15	1407	144
2	China	11	7.38	10.64	7	132	77
3	England	9	6.04	17	6	179	48
4	Brazil	8	5.37	9.45	5	101	55
5	India	6	4.02	14.33	6	164	54
6	Malaysia	6	4.02	17.78	6	232	71
7	Saudi Arabia	6	4.02	3.78	4	179	63
8	Netherlands	5	3.36	32	4	146	21
9	Romania	5	3.36	30	4	149	24
10	Kuwait	4	2.68	28.2	4	161	39

* Total link strength was automatically calculated by VOSviewer.

**Table 2:** Top 10 institutions by publication output.

Rank	Institutions	Publication Count	% of the Included Articles	Citing Articles	Times Cited	Average per Item	H-Index
1	Mercer University	5	3.36	40	49	9.8	5
2	University of North Carolina	4	2.68	122	141	35.25	4
3	King Saud University	4	2.68	65	75	18.75	3
4	Radboud University	3	2.01	157	181	60.33	3
5	University of Novi Sad	3	2.01	63	69	23	3
6	Kuwait University	3	2.01	146	147	49	2
7	Universiti Sains Malaysia	3	2.01	21	21	7	2
8	University of California System	3	2.01	125	137	45.67	3
9	Wake Forest University	3	2.01	141	142	47.33	2
10	University of Novi Sad	3	2.01	63	69	23	3

**Table 3:** Top 10 most productive authors.

Rank	Author	Publication Count	% of the Included Articles	Average per Item	H-Index	Citations	Total Link Strength *
1	Banga Ak	5	3.36	21.33	5	30	13
2	Fowler W	5	3.36	0.67	1	39	15
3	Korey A	5	3.36	5.33	2	39	15
4	Egan Kl	4	2.68	5.33	2	76	7
5	Horvat O	3	2.01	49	2	132	8
6	Kusturica MP	3	2.01	37	4	60	6
7	Bekker Cl	3	2.01	49	2	91	5
8	Egberts Tcg	3	2.01	5.5	2	11	7
9	Abahussain E	2	1.34	1	1	50	0
10	Abahussain EA	2	1.34	37	2	102	2

* Total link strength was automatically calculated by VOSviewer.

**Table 4:** Top 10 cited references of publications.

Rank	Citation Frequency	Average per Year	Title	Journal	Author
1	182	16.55	Beyond the medicine cabinet: An analysis of where and why medications accumulate	Environment International	Ruhoy IS [32]
2	177	9.83	Taking stock of medication wastage: Unused medications in US households	Research in Social and Administrative Pharmacy	Law AV [33]
3	146	8.59	Pharmaceuticals in wastewater: Behavior, preferences, and willingness to pay for a disposal program	Journal of Environmental Management	Kotchen M [34]
4	138	15.33	Disposal practices of unused and expired pharmaceuticals among the general public in Kabul	BMC Public Health	Bashaar M [35]
5	127	7.47	Household disposal of pharmaceuticals as a pathway for aquatic contamination in the United Kingdom.	Environmental Health Perspectives	Bound JP, Voulvoulis N [16]
6	118	9.83	Public practice regarding the disposal of unused medicines in Ireland	Science of the Total Environment	Vellinga A [36]
7	98	8.17	What Do Patients Do With Unused Opioid Medications?	Clinical Journal of Pain	Lewis ET [37]
8	85	10.83	Aspects Regarding the Pharmaceutical Waste Management in Romania	Sustainability	Bungau S [38]
9	84	14	Improper disposal practice of unused and expired pharmaceutical products in Indonesian households	Heliyon	Insani WN [39]
10	82	4.1	Practice and opinion towards the disposal of unused medication in Kuwait	Medical Principles and Practice	Abahussain EA [29]

**Table 5:** Top 10 most frequent author keywords.

Rank	Keywords	Occurrences	Total Link Strength *
1	medicine disposal	43	122
2	medicine waste	29	68
3	unused medicines	22	64
4	environment	12	34
5	pharmaceuticals	10	35
6	expired medicines	10	30
7	reverse logistics	9	16
8	public health	6	16
9	take-back program	6	14
10	community pharmacy	6	13

* Total link strength was automatically calculated by VOSviewer.

## Data Availability

No new data were created or analyzed in this study. Data sharing is not applicable to this article.

## Supplementary Materials

The following supporting information can be downloaded at https://www.mdpi.com/article/10.3390/healthcare13212711/s1. Table S1. Detailed findings about the knowledge (K), attitude (A), and practice (P) of stakeholders regarding drug take-back programs.
