# Supplementary material for: Bibliometric Analysis and Review of Global Academic Research on Drug Take-Back Programs
Source: Healthcare (Basel). 2025 Oct 27;13(21):2711. doi: 10.3390/healthcare13212711 (PMC12609106; doi:10.3390/healthcare13212711)
Supplement: Supplementary file 1 [file healthcare-13-02711-s001.zip › healthcare-3903400-supplementary.pdf]

Table S1. Detailed findings about the knowledge (K), attitude (A), and practice (P) of stakeholders regarding drug take-back programs.

Country/region	Participants	K	A	P	Reference
the United States	The public recruited through social media advertisements.<br>(*n*=702)	Education and provision of safe storage equipment should be designed for diverse ages, races/ethnicities, and levels of education.	Drug take-back events not hosted by law enforcement may have broader appeal, as may those led by Black or other people of color.	● Examine prescription opioid storage and participation in drug take-back events among Michigan adults, with findings identify factors associated with safe prescription opioid storage/disposal and indicate safe storage/disposal seldom occurs.<br>● Wider use of drug donation boxes may facilitate increased disposal among those who do not wish to or cannot attend take-back events.	[53]
the United States	Customers from 14 pharmacies<br>(*n*=129)	Customer awareness of dropboxes as well as knowledge about risks of improper disposal are low, however awareness was greater at pharmacies with dropboxes.	Pharmacists at dropbox locations were more consistent in their messaging to customers, more likely to recommend proper disposal methods, and more supportive of drug take-back programs.	● Further efforts focus on the increased establishment of dropboxes in pharmacies.<br>● Development of pharmacy school and employment training programs on appropriate drug disposal that include communication recommendations for pharmacists to patients.<br>● Changes to online information from federal and local agencies to improve consistency<br>● Addition of safe disposal information on medication bottles.	[24]
the United States	Drug take-back boxes	—	● Pharmacies are the most preferred take-back box location and that attitudes, subjective norms, and perceived behavioral control are statistically significant predictors of intention to use a take-back box.<br>● Results suggest that individuals are open to using take-back boxes in secure, convenient locations, but many are unaware of take-back boxes as an option for safe disposal.	—	[54]
**Country/region**	**Participants**	**K**	**A**	**P**	**Reference**

the United States	Students at the University of Vermont (*n*=358)	• Less than a quarter of students were aware of drug take-back programs (24%)<br>• Increasing awareness of, access to, and participation in pro-environment pharmaceutical behaviors, such as purchasing over-the-counter medication in smaller quantities and utilizing drug take-back programs, could minimize future pharmaceutical pollution from this population.	—	• Only 4% had ever used take-back services.<br>• The university student population may be storing a large volume of unused drugs that will require future disposal.	[55]
the United States	A convenience sample of patients at the University of Oklahoma Family Medicine Pharmacy (*n*=62)	• Interest in drug take-back programs exists, but awareness and availability of continuous programs is limited.<br>• Future studies are needed on the types of medications sent back and specific reasons for accumulation.	61% of patients reported interest in a drug take-back program.	• Most common reasons for participation in take-back programs included concern about the safety of household members, accidental or intentional ingestion, and environmental impact.<br>• Programs may be more successful if offered at no cost to patients.	[56]
the United States	Veteran patients (*n*=191)	Educate patients about the dangers of opioid use by nonprescribed others, and increase information about medication disposal options could decrease the supply of opioid medications available for misuse.	—	A majority of patients retain unused opioids, and medication sharing is common.	[37]
the United States	Patients (*n*=721)	Community-level interventions designed to improve prescription efficiency and patient awareness of appropriate disposal methods-particularly of controlled substances-are necessary to reduce the potentially harmful effects of improper disposal of unused medications.	—	—	[57]
the United States	Asian descent (*n*=62)	Patient education about tbps and their importance may be effective in increasing TBP awareness in a population with low TBP use.	—	Free disposal envelopes did not seem to be highly used within 9 months of receipt despite interest and access to uems.	[58]
**Country/region**	**Participants**	**K**	**A**	**P**	**Reference**
the United States	Drop-boxes implemented in	Counties with a higher percentage of whites, more educated	—	• A substance abuse prevention coalition, higher rates of controlled medications dispensed and prescription opioid overdose, and that	[59]

	91 (out of 100) counties(*n*=311)	residents		were Appalachian were more likely to be early adopters.<br>● A growing number of drop-boxes are being implemented in law enforcement offices and pharmacies.	
the United States	A disposal box in 2016, 2018, and 2021 among licensed pharmacies in North Carolina in 2018 (*n*=2587)	—	—	An increase in disposal boxes over time with 43 pharmacies (1.7%) in 2016, 144 (5.6%) in 2018, and 350 (13.5%) in 2021 implementing a disposal box.	[60]
the United States	Adolescent-parent dyads (*n*=243)	—	—	● Most families do not practice all recommended safe management strategies for controlled medications.<br>● Healthcare professionals should promote safe management to reduce controlled prescription medication misuse.	[61]
the United States	Phase I(*n*=238)<br>Phase II(*n*=68)	● It points to the need for public awareness and policy to reduce wastage.<br>● Pharmacists can play an important role by educating patients both on appropriate medication use and disposal.	—	Two out of three dispensed medications were unused, with national projected costs ranging from $2.4B to $5.4B. This wastage raises concerns about adherence, cost and safety.	[33]
Saudi Arabia	Pharmacy and nursing students(*n*=352)	The findings suggest that creating awareness regarding proper medicine disposal procedures among university health care students in Saudi Arabia is needed.	—	With regard to disposal, 78.9% of pharmacy students and 80.5% of nursing students reported discarding expired medicine in household garbage or flushing it down a sink or toilet. Only a small percentage returns leftover medicine to a medical store.	[71]
Saudi Arabia	Patients (*n*=1200)	● A low percentage of respondents have ever received information regarding correct medication disposal<br>● Respondents weren't aware of the consequences of keeping expired medication at home.	—	1、 Over half of the respondents store antibiotics in their households.<br>2、 The awareness of proper and safe drug disposal among the Saudi population is quite low making it a priority of concerned authorities to implement educational programs.	[72]
**Country/region**	**Participants**	**K**	**A**	**P**	**Reference**

the United States	Users(n=35) and non-users (n=20) of a medication take-back program located at a community pharmacy in Texas.	—	● All users viewed the medication take-back program as a valuable service, while nearly all (90%) non-users viewed the program as a potentially valuable service.<br>● In comparison to non-users, users were significantly older, had more favorable perceptions about paying for the service, and were more likely to choose a pharmacy that provides the service.	The majority of users were participating in a take-back service for the first time.	[62]
the United States	Participants completed all vignette drug disposal scenarios (n=1006)	—	Programs that provide disposal resources directly to the patient at no cost with their prescription are likely to optimize willingness to dispose.	Findings support the FDA's plan for a REMS program requiring pharmacies to distribute mail-back envelopes to patients when dispensed opioids.	[63]
the United States	Served community members (n= 31)	—	—	● Pharmacists can have a vital role in preventing diversion of opioid analgesics and associated consequences.<br>● There is a need to expand disposal boxes at pharmacies to increase community member accessibility and use.<br>● Future research is needed to determine the cost-effectiveness of expanding the scale of disposal box implementation in community pharmacies.	[64]
the United States	Residents in southern California (n=1005)	—	● The results of a contingent valuation question indicate a substantial willingness to pay a surcharge on prescriptions to support the establishment of a pharmaceutical disposal program.<br>● Also find that respondents are likely to participate in a disposal program.	● While disposal of unused medications through the trash and toilet/sink is the most common practices, respondents that are aware of the issue are more likely to return pharmaceuticals to a pharmacy or drop them off at a hazardous waste center.<br>● Assuming that the program is based on drop-off locations at local pharmacies, approximately 70 percent of the respondents would be very likely to return their unwanted or expired medicines.	[34]
China	Patients with a median age of 62 years(n=221)	—	—	● A significant proportion of Chinese patients with cancer exhibit unsafe practices in the storage, disposal, and use of opioids.<br>● The study highlights an urgent need for implementing routine education programs and drug "take-back" initiatives to improve opioid-related practices.	[65]
**Country/region**	**Participants**	**K**	**A**	**P**	**Reference**
China	Residents from China(n=366)	—	● Return intention plays a mediating role in the positive effect of consequences awareness of the public environment on proper return behavior.	The direct effect of residents' consequences awareness of public environmental awareness on the proper medicine return	[66]

Country/region	Participants	K	A	P	Reference
			● Personal norms and return intention play a chain mediating role in the positive impact of consequences awareness of the public environment on proper return behavior.<br>● Personal health awareness moderates the chain mediation path by strengthening the positive effect of return intention on proper return behavior.	behavior is not significant	
China	Community residents<br>(*n*=613)	● The knowledge of Guangzhou residents regarding household expired drugs disposal were not ideal.<br>● To improve the resident's awareness about family expired drugs disposal	The attitude of Guangzhou residents regarding household expired drugs disposal were not ideal.	● The practice of Guangzhou residents regarding household expired drugs disposal were not ideal.<br>● Ensure the financial support for the recycling process, establish an accessible and acceptable recycling point, and introduce relevant laws and regulations for the long-term mechanism.	[67]
China	Households<br>(*n*=625)	● Despite some attention being given to the location of storage for home medicines, considerably more information is needed to improve awareness.<br>● Some attention had been paid to elimination and recycling mechanisms but similarly, increased awareness is needed.	—	A major improvement in the awareness of correct storage conditions of medicines for home use can be realized by increased education, and highlighting the importance of correct medicine storage, disposal methods and usages	[68]
China	Valid household<br>(*n*=459)	—	● Subjective norm and recycling attitude directly influences HPW recycling intention, except perceived behavioral control.<br>● As for the newly added variables, economic incentive, information publicity, and trust in manufacturers noticeably influence HPW recycling intention, in contrast,	Providing guidance on increasing the involvement of households in recycling activity for designing and modifying the HPW reverse logistics.	[69]

Country/region	Participants	K	A	P	Reference
			the trust in government and retailers is not significant in predicting HPW recycling intention. ● Information publicity can exert an indirect effect on recycling intention through subjective norms and recycling attitudes.		
China	Respondents (n=1865)	—	The level of support for a future waste medicine take back scheme was less than 40% being wholehearted supporters.	● Observations also suggest appreciable noncompliance and consumption of unused medicines following self-diagnosis of symptoms. ● Due to lack of consumption data, pharmaceutical waste handling infrastructure and medication compliance.	[70]
Saudi Arabia	Saudi adult (n=337)	Increasing awareness through education programs about proper disposable guidelines is necessary for controlling the medication wastage.	—	The disposable practice among the Saudi community was inadequate.	[73]
Saudi Arabia	Subjects (n=360)	There was no significant association between the community pharmacists' age group and years of practice as community pharmacists with either the awareness of unused medication disposal on environmental hazards, or the beliefs about the appropriate location for collecting unused drugs ($p > 0.05$).	The awareness and proactive accountable responsibility, along with community pharmacists' belief of appointing pharmacies to collect unused drugs, strongly support the institution of drug take-back programs.	More than 70% returned the unused drugs to the pharmaceutical distributors.	[74]
**Country/region**	**Participants**	**K**	**A**	**P**	**Reference**
Malaysia	Respondents (n=204)	In order to implement planned programs for proper collection and destruction of waste medication, a plan is needed to enhance public knowledge on the impacts of improper medication waste disposal on the environment	● Attitudes, personal norms, perceived busyness, and perceived behavioural control have significant effects on intention to dispose of unused medicines. ● Perceived convenience moderates the impacts of attitude, personal norms, and perceived behavioural control on intention to dispose of unused medicines. ● The importance of integrating additional variables into the TPB to enhance its explanatory power in predicting	—	[75]

			behavioural intention.		
Malaysia	Participants (*n*=1184)	—	Unused medicines are associated with people who use medicines for acute illnesses, pay for their medication, and are willing to participate in an MRP	• The prevalence of unused medicines and their improper disposal were high in Malaysia.<br>• Rationale prescription and optimal dispensing practice, together with a broader MRP facilities coverage, could reduce unused medicine possession.	[76]
Malaysia	Households (*n*=103)	Most of the participants were aware that pharmaceutical waste can have an adverse impact on the environment and public health.	—	• The study concludes that while the respondents were aware of the adverse impact of HPW, their practices to ensure proper disposal is discouraging.<br>• There is a need for effective unwanted medicines return-back programme as a more prudent disposal method of HPW to avoid any risk to the environment or human health.	[23]
Brazil	Medicine consumers (*n*=204)	—	—	To encourage the proper disposal of medicines, it present options for regulations and persuasive marketing practices comprising communication and short-term incentives.	[81]
**Country/region**	**Participants**	**K**	**A**	**P**	**Reference**
Malaysia	Community pharmacists (*n*=18)	—	Pharmacists have positive perceptions of the safe disposal of medicines.	Pharmacists mentioned that medicine returns to service in community pharmacies are not common due to a lack of facilities in the management of unwanted, expired, and returned medicines. As such pharmacists have suggested a few ways to minimize medicinal wastage.	[77]
Serbia	Families (*n*=230)	—	—	Public services in Serbia, including government and health sectors, need to be more proactive about educating people on how to store and dispose medications, as well as finding a way for implementation of	[78]

				the law on medications wastage destruction.	
Serbia	Families<br>(*n*=1008)	This study revealed that there were relatively large quantities of expired medications in Serbian households, with a high prevalence of antibiotics for systemic use, anti-inflammatory and antirheumatic products, and medications for alimentary tract and metabolism.	—	—	[79]
Serbia	Pharmacies<br>(*n*=683)	—	—	● The pharmacies have not started to implement their legal obligation of collecting pharmaceutical waste from the citizens yet, at least not in the full range.<br>● The development and practical implementation of adequate procedures and facilities for the disposal of this type of waste represents the key step towards the decrease of pharmaceutical waste in the environment.	[80]
**Country/region**	**Participants**	**K**	**A**	**P**	**Reference**
Serbia	Patients<br>(*n*=800)	—	● Approximately 80% of surveyed respondents are very or somewhat likely to participate, however less than half of the respondents are willing to pay for the collection of their unused medicines.<br>● The factors that influenced willingness to participate are environmental awareness and income, while the factors affecting willingness to pay, are previously received advice about proper disposal, education level, number of unwanted medicines in the household and gender.	The majority of Serbian people dispose unused medicines improperly, mostly into household garbage. Well-organized and easily accessible collection programs are essential in order to enable the general public to return unused medicines for proper disposal.	[41]
India	Urban residents<br>(*n* =238)	—	Personal norms and attitudes were found to significantly mediate the effect of the antecedents on return intention.	The findings suggest that policies enhancing awareness and promoting altruistic values can significantly boost REOUM efforts, contributing to sustainable medicine consumption practices.	[86]

Australia	Respondents (*n*=4302)	Limited awareness could lead to environmental or public health risks, and targeted information campaigns are needed.	Respondents were largely unaware of the RUM Project, yet were willing to use it once informed.	Two-thirds of respondents disposed of medicines with the household garbage and approximately one-quarter poured medicines down the drain.	[90]
India	Healthcare professionals (*n*=300)	Nurses (76%) had significantly (P < 0.01) better knowledge over doctors regarding the methods of drug disposal, whereas both doctors (63%) and nurses (64%) knew significantly (P < 0.05) more than pharmacists about type of formulations not to be incinerated.	• A large proportion of hcps had a positive attitude toward checking the expiry date of medicines (96%), and a significant proportion of doctors and nurses believed that drug disposal is everyone's responsibility.<br>• Nearly half (44%) of all hcps agreed that there existed a system for removing expiry and unused medicines from the inventory.	Nurses (59%) had better (P < 0.01) practice than pharmacists regarding the disposal of expired medicines.	[87]

**Country/region**	**Participants**	**K**	**A**	**P**	**Reference**
Brazil	Respondents (*n*=952)	—	—	The findings emphasize the significance of user cooperation and provide insights for the development of effective strategies and policies to address pharmacopollution.	[82]
Brazil	Pharmacists (*n*=630)	It is important to increase knowledge through professional training and further education programs.	—	A small number of pharmacists always orient customers on the proper disposal that should be followed for unused and expired household medications, prioritizing their return to a pharmacy.	[83]
Brazil	Pharmacists (*n*=161)	35% have partial knowledge of the postconsumer reverse logistics of their place of work and 16.8% have complete knowledge of the environmental harm resulting from the contamination of medicinal waste.	—	The pharmacists reported (10%) that there should be an educational plan for clients, and 50% of them agreed that incentives and disclosure, regarding the importance of reverse logistics for medicines, should be provided to consumers.	[84]
the United Kingdoms	UK adults(*n*=663)	—	—	The results suggest people use different mental models for each destination with disposal in sink/toilets and bins considered quicker and safer in the presence of children or for those feeling anxious.	[85]
the United Kingdoms	Returns(*n*=114)	The results from this pilot study indicate that unused medicines impose a significant financial burden on the National Health Service as well as a social burden on the	—	—	[28]

		United Kingdom population.			
India	Respondents (*n*=190)	—	—	The findings of this research priority-setting exercise can help to guide research for the development of policy-relevant and novel interventions to improve SRUM in India.	[49]
Kuwait	Patients or their family members (*n*=300)	—	—	Almost half of the respondents (45.4%) obtained medicines on prescription more than 3 times a year and almost all had unwanted medicines in the home. Reasons for possessing unused medication were mostly due to a change of medication by the doctor (48.9%), or self-discontinuation (25.8%).	[29]
Kuwait	Households (*n*=200)	—	—	No medicines were collected from the 200 households participating in the municipal collection scheme in spite of 45% of respondents agreeing that this was an appropriate method of disposal.	[88]
**Country/region**	**Participants**	**K**	**A**	**P**	**Reference**
Kuwait	Pharmacists (*n*=144)	—	● Even though the current practice of disposal by majority of pharmacists is inappropriate, they are aware of the damage and acknowledge their responsibilities toward environment protection.<br>● Majority of pharmacists support the idea of having the government pharmacies as collection points for safe disposal of unwanted medicines in Kuwait.	Throwing ums in the trash was the main method of disposal by majority of the respondents (73%). Only 23 pharmacists disposed ums according to the guidelines of Ministry of Health, Kuwait (MOH).	[89]
Australia	Households (*n*=166)	—	—	Although accidental ingestion in children and pets and decreased efficacy were recognised health risks, this did not always translate to appropriate storage, usage or disposal practices.	[4]
Nigeria	Students (*n*=930)	A knowledge gap was revealed among the respondents regarding the safe disposal of unused medications.	—	● Despite the presence of knowledge and awareness, these do not necessarily translate into good disposal practices.<br>● This call for strategies to overcome identified barriers, with the aim to bridge the knowledge-practice gap and promote safe disposal of unused medication.	[91]

Nigeria	Households (*n*=130)	Only 10.5% (n= 11) of respondents knew how medicines were properly disposed.	—	While most households store their medicines appropriately, majority of them adopt poor disposal methods for medicines they no longer need in their homes.	[92]
Jordan	Participants (*n*=1092)	—	—	● A high percentage of the Jordanian population improperly handles their unused, leftover or expired medications. 2、More restrictions are needed on the prescribing and dispensing of medications by the authorities concerned and there is a need to establish public guidelines regarding the use and correct disposal of pharmaceuticals.	[93]
**Country/region**	**Participants**	**K**	**A**	**P**	**Reference**
Jordan	The Ministry of Environment, pharmaceutical manufacturers, and pharmaceutical warehouses.	The study revealed that there is a lack of awareness among the public toward household pharmaceuticals.	On the other hand, the public and the Ministry of Environment were supportive of establishing the program.	The study recommends developing a comprehensive legislation and regulatory framework for household pharmaceuticals' recovery, establishing a monitoring entity, securing funds, and implementing a long-term awareness and education plan to support the establishment of a pharmaceutical take-back program in Jordan.	[94]
Romania	People(*n*=739)	The results revealed the level of public awareness regarding the legal disposal methods and destruction of the pharmaceutical wastes	—	The urgent need of public information campaigns (including educational campaigns for implementation of cost-effective and optimum unused pharmaceutical disposal strategies) that are nowadays totally insufficient and could be performed	[95]
Romania	Pharmacists (*n*=521)	—	Pharmacists refuse to collect the pharmaceutical waste	The management of drug-based waste generated by the population is at the beginning	[38]
Germany	Healthcare institutions randomly selected in Ghana and the general public	—	—	Over 75% disposed of pharmaceutical waste through the normal waste bins which end up in the landfills or dump sites.	[96]

	(*n*=5)				
Sweden	The Pharmaceutical Industry AB (LIF) has together with the Swedish retail chain Apoteket AB	Environmental concerns are getting more important than security concerns as a reason for returning unused medicines to a pharmacy and a growing fraction is worried about the environmental impact of pharmaceuticals.	—	An increasing number of the Swedish population does return unused medicines to a pharmacy for correct disposal.	[97]
**Country/region**	**Participants**	**K**	**A**	**P**	**Reference**
Sweden	Swedish pharmacies	—	—	Hoarding or over-supply of prescribed medicines may explain a large part of the volume of medicines that remain unused. Iming to reduce waste of prescribed medicines ought to focus on those patients who contribute to a substantial part of all unused medicines.	[31]
Ireland	Individuals (207 in Galway and 191 in Cork) (*n*=398)	There is little awareness among members of the public regarding appropriate ways to dispose of unused medicines.	—	Environmentally inappropriate disposal methods were through general waste disposal and via the sewage system.	[36]
Indonesian	Respondents (*n*=497)	● A significant percentage of them never received information about proper medication disposal practice (79.5%).<br>● More than half of the respondents were unaware that unsafe medication disposal practices could harm the environment and population health (53.1%).	—	Disposal of unwanted pharmaceutical products through environmentally unsafe route was prevalent among the respondents.	[39]
Tanzania	Randomly selected household members (*n*=359)	Majority of respondents (273 (76%) were aware that improper disposal of expired medications are detrimental to human health and environment in general.	—	● Improper disposal of unused and expired medications at household level was a common practice in the study area.<br>● Tailor-made interventions by the Food and Drugs Authority (FDA) and other national	[100]

				as well as local stake holders are urgently needed to address the situation.	
Ethiopia	Health professionals (n=135)	—	—	Determinants of pharmaceutical waste management were being male, receiving training, writing label, storage of segregated waste in dispensing unit and the presence of disposal plan.	[103]
**Country/region**	**Participants**	**K**	**A**	**P**	**Reference**
Thailand	Structured questionnaires (n=400)	Most of them showed a low degree of medication management, pointing to the need for targeted interventions.	This study identifies the factors that have a positive influence on proper medication disposal practices, such as age, level of education, attitude, and perception.	In terms of policy, the study highlights the need for comprehensive interventions, including medication take-back systems, infrastructure development, and educational initiatives.	[98]
Poland	Polish residents (n=981)	—	It is worth mentioning that there is an association between a survey participant's awareness of the proper methods for disposing of pharmaceuticals and their choice of a disposal method.	We recommend education programmes for pharmacists to provide them with information about what sort of drug disposal information their consumers need.	[99]
Nepal	Adults(n=400)	—	● Analysis of willingness to follow take-back program with the techniques of support showed significant relationship with the establishment of collection center and participation on seminar (P value < 0.01).<br>● Most participants were interested to support take-back, if implemented in their community but main constraint was the budget.	Take-back concept could be initiated and implemented on government funding or other sources.	[101]

South Africa	Respondents (*n*=286)	—	• The respondents generally exhibited strong disagreements with environmentally unfriendly and health-threatening disposal practices.<br>• Moreover, most participants were willing to return expired medicines to pharmacies (40.7%, n = 151), whereas only 8.6% (n = 32) opposed this solution.<br>• The study found that education attainments significantly influenced the willingness of respondents to return pharmaceutical wastes.	There were no significant differences amongst respondents in terms of waste disposal practices. The findings suggest the need for targeted efforts to bring about sustainable waste management at a household level.	[111]
**Country/region**	**Participants**	**K**	**A**	**P**	**Reference**
Cyprus	Citizens in Cyprus (*n*=184)	—	More than 55% of the participants indicated that they will follow a specific waste management program if existing in place.	The main disposal method of unused or expired medicines and drugs is in household waste followed from the sink and the toilet.	[104]
Algeria	Students(*n*=328)	—	The results revealed that perceived behavioral control (PBC), start-up self-efficacy, drug waste collection-related knowledge and subjective norms have a positive significant effect on the intention to create a start-up to collect unwanted drugs.	—	[105]
Afghanistan	The general public (*n*=301)	Almost entire sample (98%) felt that improper disposal of unused and expired medicines can affect the environment and health.	Majority of respondents held government responsible for creation of awareness for proper medicine disposal.	Majority (95.3%) of the respondents' stored medicines at home. 77.7% of the respondents discarded the expired medicines in household trash.	[35]
Ghana	Respondents (*n*=131)	—	—	• The predominant method of disposal of expired/unwanted medicines by community pharmacies and their clients was via the general-purpose bin.<br>• Implementation of interventions such as take back programmes that will enhance proper disposal of expired and	[106]

				left over medicines should be initiated.	
the United Arab Emirates	Pharmacists (*n*=370)	The most cited barriers were a lack of public awareness about the risks of improper medication disposal (88.9%), lack of time due to workload (82.4%), and lack of training and education (78.1%).	Most pharmacists believed in collaborating with public health agencies to promote medication disposal awareness (97.6%) and developing national guidelines (97.6%).	Factors such as pharmacist age, type of pharmacy, country of education, pharmacy location, employment status, and years of experience were significantly associated with pharmacists' level of involvement in promoting safe medication disposal practices.	[107]
**Country/region**	**Participants**	**K**	**A**	**P**	**Reference**
Palestine	Practicing pharmacists (*n*=500)	A total of 48.5% of the participants indicated that a lack of education and awareness on the issue of getting rid of unused drugs constitutes a challenge to the safe disposal of medicines, and 66% of them said that a lack of law enforcement constitutes another challenge.	● A total of 61.3% of pharmacists agreed and 26% strongly agreed that unsafe disposal of drugs negatively affects the environment.<br>● A total of 93.3% supported distributing educational brochures, and 92.8% supported placing special containers in every pharmacy to collect unwanted drugs.	Current data emphasize the issue of improper disposal of medicine in Palestine and the need for improved education among healthcare workers.	[108]
Croatia	Pharmacies (*n*=210)	Advertising of the service may increase awareness of the importance of proper disposal of unused medicines.	—	Governmental bodies should examine current legislation regulating pharmaceutical waste disposal, particularly financial responsibility for providing the service, in order to increase pharmacies' compliance.	[109]
Pakistan	The pharmacy students(*n*=614)	The knowledge was significantly associated with the gender ($p = 0.017$), year of study ($p = 0.001$) and institutes ($p = 0.001$) of respondents.	—	● A gap exists between their awareness and practice of disposing of medicines.<br>● Ministry of Education and Ministry of Health, should provide appropriate education on the safe disposal of medicines that might require curriculum revision.	[110]

Eritrea	Participants (*n*=327)	Unnecessary storage and improper disposal of household unused/expired medicines along with inadequate knowledge on disposal mechanisms were common in households of Asmara.	Participants had a satisfactory willingness to participate in a household medicines take-back system, with a mean attitude score of 16.89/20 (95% CI: 16.45-17.29).	The most commonly used disposal practices were throwing with household garbage (65.6%), followed by dumping under soil (38.7%) and flushing down the toilet/sink (15.2%).	[111]
**Country/region**	**Participants**	**K**	**A**	**P**	**Reference**
Canada, the United States, and Europe	Interviewees (*n*=27)	—	They believed that advanced wastewater treatment technology, education of medical professionals to reduce overprescription, pharmaceutical-return programs coupled with public education, and requirements for all municipalities to have a minimum of secondary wastewater treatment were the most effective management strategies to reduce the environmental impacts of pharmaceuticals.	—	[30]
Gulf Cooperation Council countries	Pharmacists (*n*=277)	—	—	Many waste-minimising activities were undertaken by pharmacists in the prescribing, dispensing, and leftover stages.	[112]
